# The off-label use of targeted therapies in sarcomas: the OUTC’S program

**DOI:** 10.1186/1471-2407-14-870

**Published:** 2014-11-24

**Authors:** Lauriane Eberst, Claire Cropet, Axel Le Cesne, Patricia Pautier, Nicolas Penel, Antoine Adenis, Christine Chevreau, Jacques-Olivier Bay, Olivier Collard, Didier Cupissol, Florence Duffaud, Jean-Claude Gentet, Sophie Piperno-Neumann, Perrine Marec-Berard, Emmanuelle Bompas, Antoine Thyss, Loic Chaigneau, Philippe Cassier, François Bertucci, Jean-Yves Blay, Isabelle Ray-Coquard

**Affiliations:** Centre Léon Bérard, 28 rue Laennec 69 373, LYON CEDEX 08, France; Université Claude Bernard Lyon 1, Lyon, France; Institut Gustave Roussy, Villejuif, France; Centre Oscar Lambret, Lille, France; Institut Claudius Regaud, Toulouse, France; Centre Jean Perrin, Clermont-Ferrand, France; CHU Estaing, Clermont-Ferrand, France; Institut de Cancérologie Lucien Neuwirth, Saint-Priest-en-Jarez, France; Institut régional du Cancer, Montpellier, France; CHU La Timone, Marseille, France; Institut Curie, Paris, France; Centre René Gauducheau, Nantes, France; Centre Antoine Lacassagne, Nice, France; CHU Jean Minjoz, Besançon, France; Institut Paoli-Calmettes, Marseille, France

**Keywords:** Off-label, Sarcoma, Targeted therapy, Register, Tumor board

## Abstract

**Background:**

Few targeted therapies (TTs) are registered for sarcoma treatment despite numerous phase II studies and yet there are potential treatment options for patients after standard treatment escape. The French Sarcoma Group - Bone Tumor Study Group (GSF-GETO) created a national registry to evaluate the outcome of patients treated with off-label TTs.

**Methods:**

Every consecutive sarcoma-patient receiving an off-label TT outside a clinical trial was included. The objective was to describe this patient efficacy and safety data in routine practice.

**Results:**

From October 2008 to October 2011, 249 patients in 24 centers received 278 treatment lines with TTs. Twenty-five histological subtypes were included: most frequent were leiomyosarcoma (*n* = 48, receiving sorafenib in 63%, and sunitinib in 27%), GIST (*n* = 39, receiving sorafenib in 79%), and angiosarcoma (*n* =18, receiving sorafenib in 78%). The overall response rate to TTs was 15% (95% CI [10,6-20,2]), the disease control rate at 2 months was 59%. The median progression-free survival was 4,1 months (IC 95% [3,2-4,8]). Three complete responses were observed. No toxic death occurred, grade 3 and 4 toxicities were reported in 74 (27%) and 14 patients (5%) respectively.

**Conclusion:**

Off-label TTs can be used for sarcoma patients in routine practice with an acceptable toxicity profile and efficacy similar to that reported in non-randomized clinical trials.

**Electronic supplementary material:**

The online version of this article (doi:10.1186/1471-2407-14-870) contains supplementary material, which is available to authorized users.

## Background

Soft tissue sarcomas (STS) are rare tumors with an incidence of 6/100000/year [1, 2]. Patients with metastatic sarcoma have a poor prognosis and their median overall survival (OS) doesn’t exceed 18 months [3]. More than 50 histological subtypes have been identified and their diagnosis often requires an expert review [4]. In the last two decades, progress in molecular biology have lead to a better understanding of sarcomas biology and ontology, though with limited therapeutic implications since the standard treatment of metastatic sarcoma remains chemotherapy, regardless of specific molecular alterations [5]. The registered cytotoxic agents are anthracyclines, ifosfamide, dacarbazine and trabectedin. The gemcitabine-docetaxel and gemcitabine-dacarbazine combinations have also been reported as active treatments in some histological subsets [6–9]. These regimens are cited as options in clinical practice guidelines from the European Society for Medical Oncology (ESMO) [10] or the National Comprehensive Cancer Network (NCCN) [11].

However, the success of imatinib in gastro-intestinal stromal tumors (GISTs) showed the relevance of guiding treatment to specific molecular alterations [12] and similar observations have been obtained in other rare histological sarcoma subtypes [13, 14], leading to the approval of several targeted therapies (TTs). Imatinib, a multi-target tyrosine-kinase inhibitor, and aside form GIST has been shown to have activity in several sarcoma subtypes where it interferes with key oncogenic drivers: the protein product of the COL1A1-PDGFB fusion transcript in dermatofibrosarcoma protuberans (DFSP) [15, 16] and the colony-stimulating factor 1 in pigmented vilonudular synovitis [17]. Its mechanism of action remains unclear in aggressive fibromatosis (AF), but several phase II trials confirmed its activity [18, 19]. Denosumab has been approved for the treatment of giant-cell tumor of bone, it blocks tumor-induced bone destruction by inhibiting RANK-RANK-ligand interaction [20]. However, there is no known actionable molecular alteration in the vast majority of sarcomas. Overall sarcomas are difficult to study because of their heterogeneity. This may explain why several phase II trials testing TTs for STS (imatinib [21], sorafenib [22], sunitinib [23]) haven’t lead to registration or to phase III trials despite signs of potential activity. Pazopanib was recently approved by both the Food and Drug Administration and European Medecines Agency following the positive results of the Palette study [24]. Ridaforolimus on the other hand was not approved despite a positivity of the SUCCEED trial [25].

It’s not unusual, according to medical practice analysis, that patients with advanced sarcoma receive four or more lines of treatment [26], TTs are then interesting treatment options when patients experience disease progression despite standard treatments. Because several commercially available agents had shown signs of activity in sarcomas in retrospective and prospective phase II studies, the French Sarcoma Group setup a national registry in 2008. This registry, called OUTC’S (Observatoire de l’Utilisation des Thérapies Ciblées dans le Sarcome/Observatory for the use of targeted therapies in sarcomas), aimed to collect in a prospective manner all medical data regarding the use of off-label TTs in sarcoma and assess their activity in routine practice. Off-label prescription is authorized in France for rare disease under control of experts, based on published data reporting potential activity. The objectives of this study were to analyse the activity and toxicity of TTs in sarcoma and eventually identify specific subsets of patients responding to a particular TT in a given histological subtype. This registry could hence be useful for a better understanding of sarcoma and to develop new therapies for patients.

## Methods

### Patients/registry

Patients with the following criteria were included: sarcoma with confirmed histological diagnosis, not amenable to curative treatment, treatment in France with non-approved TT (i.e. a drug interfering with a specific molecular target) and outside any clinical trial. GIST patients treated with imatinib or sunitinib were excluded (these drugs had already been approved). In spite of the recent approval of imatinib for DFSP and because more clinical data was needed, patients treated with imatinib were included, as well as bone sarcoma patients treated with zoledronic acid, considering promising results of this treatment as TT in preclinical models of bone sarcomas [27, 28]. As the study was not interventional, formal written consent was not required by French law. However, patients were informed and gave oral consent for data collection and use of clinical data for research purposes. Children could be included with their parents’ consent. All patients received a detailed information letter and had the opportunity to withdraw their consent at any time.

### Competent authorities approval

All data was collected by the coordination center (Centre Léon Bérard, Lyon) after approval of the Centre Léon Bérard Clinical Trial Review Committee and the French data protection authority (CNIL). The study had to be approved by the Multidisciplinary Sarcoma Board (MSB) of all participating centers (Additional file 1: Participating centers) according to the French Cancer Plan recommendations (2003–2007). Most decisions of off-label TT treatment were made after patients were discussed at a MSB, as defined by the French SARComa NETwork (Netsarc [29]) including at least three experts. Eighteen comprehensive cancer centers and eight university hospitals (all members of the GSF-GETO) participated and included all consecutive patients if they met inclusion and exclusion criteria (two centers did finally not include any patient). Once a patient was registered, follow-up was established every two months by the coordination center.

### Data collection and study endpoints

The primary objective of this study was to describe the efficacy of off-label TTs in sarcoma-patients. Secondary objectives included characterization of toxicity, feasibility of such off-label prescription in routine practice and description of unique exceptional responses in particular sarcoma subtypes. Data was collected from clinical files. Efficacy endpoints included response rate (RR) to a given TT, overall response rate to treatment (ORR) (i.e. rate of complete and partial responses [CR, PR] according to RECIST criteria [30]), disease control rate at two, four and six months (i.e. rate of CR and PR and stable disease [SD] as best overall response), PFS under treatment and OS.

### Statistical methods

The ORR was calculated with its 95% confidence interval (CI). PFS was calculated from the beginning of TT to the date of event, defined as the first documented progression under treatment or death due to any cause under treatment. Patients who did not experience an event were censored at the date of treatment and in cases of premature treatment discontinuation, before the end of follow-up or at the date of last contact for patients still under treatment. OS was calculated from the date of diagnosis until the date of death due to any cause and censored at the date of last contact for patients alive. PFS and OS were estimated by the Kaplan-Meier method. Safety evaluation was based on the frequency and severity of toxicities, graded according to the Common Terminology Criteria for Adverse Events (AE) [31].

Patients could be included more than one time in the registry when receiving consecutive lines of TTs. All analyses were performed on total number of treatment lines (expressed as ‘patients’ when described), except for data regarding OS, which was analysed on the total number of patients included at least once in the study. For patients included several times, OS was calculated as the time between initial diagnosis and date of last follow-up for the latest treatment. The research database was locked for the statistical analysis in October 2011. The analysis is descriptive. All expressed CIs are two-sided.

## Results

### Patients characteristics

From October 2008 to October 2011, 249 patients in 24 institutions were registered and received a total of 278 lines of treatment (21 and 4 patients received two and three successive lines of TT respectively). The population characteristics are presented in Table 1. The median time from sarcoma diagnosis to treatment in the registry was 3.5 years (range 15 days to 32 years). The median number of previous systemic therapy (chemotherapy) was three (range 0–9). Fifteen patients received a TT as their first line of systemic therapy. In all cases these patients had a sarcoma subtype or connective tissue tumor with no other standard option (AF, angiosarcoma, chordoma…), and were treated with imatinib, sorafenib, or mTOR inhibitors. First line treatment is detailed in Additional file 2: Table S1). A median number of three lines of chemotherapy (range 0–9) was administered before starting TT. At the time of initiation of TT, 70% of patients had a documented RECIST progressive disease on their previous treatment. The decision to use off-label TT was made after discussion in MSB for 203 patients (76%).

**Table 1:** **Population characteristics**

	Total*	
	N = 278	
Sex		
Male (%)	153	(55.0)
Female (%)	125	(45.0)
Age at initial histological diagnosis (years)		
Mean (SD)	42.7	(17.8)
Median (min-max)	45.0	(6–81)
Unknown/missing data (%)	1	(0.4)
Age at beginning of TT (years)		
Median (min-max)	49.0	(8–81)
≤18 years (%)	16	(5.8)
≥70 years (%)	36	(12.9)
Tumor localization		
Abdomen (%)	60	(21.6)
Lower limb (%)	53	(19.2)
Pelvis (%)	37	(13.3)
Thorax (%)	36	(12.9)
Axial skeletton (%)	26	(9.3)
Upper limb (%)	24	(8.7)
Head/neck (%)	20	(7.2)
Retroperitoneum (%)	20	(7.2)
Unknown (%)	2	(0.7)
Histological subtype		
GIST (%)	39	(14.1)
Leiomyosarcoma (%)	36	(13.0)
Angiosarcoma (%)	18	(6.5)
Unclassified sarcoma (%)	15	(5.4)
Chordoma (%)	15	(5.4)
Osteosarcoma (%)	15	(5.4)
Synovialosarcoma (%)	15	(5.4)
Ewing/PNET (%)	14	(5.1)
Chondrosarcoma (%)	12	(4.3)
Uterine leiomyosarcoma (%)	12	(4.3)
Liposarcoma (%)	12	(4.3)
Solitary fibrous tumor (%)	10	(3.6)
Epithelioid sarcoma (%)	9	(3.2)
MPNST (%)	8	(2.9)
ASPS (%)	8	(2.9)
DSRCT (%)	6	(2.1)
Aggressive fibromatosis (%)	6	(2.2)
DFSP (%)	5	(1.8)
PEComa (%)	4	(1.4)
Rhabdomyosarcoma (%)	3	(1.1)
Kaposi sarcoma (%)	1	(0.4)
Low grade endometrial stromal sarcoma (%)	1	(0.4)
Phyllode tumor (%)	1	(0.4)
Other (including benign tumors) (%)**	13	(4.7)
Tumor grade		
Unknown (%)	93	(33.4)
Non evaluable (%)	27	(9.7)
Grade I (%)	22	(13.9)
Grade II (%)	38	(24.1)
Grade III (%)	98	(62.0)
Metastatic phase at diagnosis		
Yes (%)	96	(34.5)
Number of lines of chemotherapy before TT (N = 278)		
0(%)	31	(11.2)
1(%)	38	(13.7)
2(%)	47	(16.9)
3(%)	71	(25.5)
≥4(%)	91	(32.7)

*number of lines of treatment.

**other histologies: angiomyolipoma (n = 2), ependymoma (n = 2), nephroblastoma (n = 1), medulloblastoma (n = 1), inflammatory myofibroblastic tumor (n = 1), chemodectoma (n = 1), giant cell tumor of bone (n = 1), malignant schwannoma (n = 1), perineurioma (n = 1), hemangioperycitoma (n = 1).

ASPS: alveolar soft parts sarcoma.

DFSP: dermatofibrosracoma protuberans.

DSRCT: desmoplastic small round cell tumor.

GIST: gastro-intestinal stromal tumor.

MPNST: malignant peripheral nerve sheath tumor.

SD: standard deviation.

TT: targeted therapy.

### Efficacy of off-label targeted therapy

Sorafenib was used for 125 patients (45%) as a single agent (*n* = 120) or with dacarbazine (*n* = 3), metformine (*n* = 1), or paclitaxel (*n* = 1). Sunitinib was used for 67 patients (24%), including one case of sunitinib combined with cyclophosphamide. Sirolimus was given to 25 patients (9%), in most cases in combination with cyclophosphamide (*n* = 18). Imatinib was used as single agent in 23 patients (8%), and in combination with everolimus (*n* = 3). Other TTs were everolimus (*n* = 10), bevacizumab (*n* = 9), temsirolimus (*n* = 4), nilotinib (*n* = 3), pazopanib (*n* = 2), zoledronic acid (*n* = 2), enzastaurin (*n* = 2), crizotinib, cetuximab, erlotinib, masitinib, panobinostat, and deforolimus (one patient each). Table 2 describes the most frequent TTs by major subtypes.

**Table 2:** **Targeted therapy by histotypes**

Targeted therapy	N	%	Histotype 1	n (%)	Histotype 2	n (%)	Histotype 3	n (%)	Histotype 4	n (%)	Histotype 5	n (%)
**Sorafenib (1)**	125	45	GIST	31 (25)	LMS	22 (18)	AS	14 (11)	Uterine LMS	8 (6)	Liposarcoma	8 (6)
**Suntinib (2)**	67	24	LMS	9 (13)	Ewing	8 (12)	SS	8 (12)	Unclassified S	8 (12)	Uterine LMS	4 (6)
**Imatinib**	23	8	Chordoma	8 (35)	AF	4 (17)	DFSP	4 (17)	Epithelioid S	2 (9)	─	─
**Sirolimus-cyclophosphamide**	18	6	OsteoS	8 (44)	ChondroS	5 (27)	AS/chordoma/lipoS/Ewing/SFT	1 each (6)	─	─	─	─
**Everolimus (3)**	10	4	GIST	3 (30)	LMS	3(30)	KS/MPNST/SS	1 each (10)	Other	1(10)	─	─
**Bevacizumab (4)**	9	3	Other	5 (56)	MFST	2 (22)	AS	1 (11)	Epithelioid S	1 (11)	─	─
**Sirolimus alone**	5	2	OsteoS	2 (40)	PEComa	1 (20)	other	1 (20)	─	─	─	─

Targeted therapies with less than 5 patients are not described in this table.

(1) alone in 120 cases, combination in 5 cases.

(2) alone in 66 cases, combination in 1 case.

(3) alone in 7 cases, combination in 3 cases.

(4) alone in 3 cases, combination in 6 cases.

AF: aggressive fibromatosis.

AS: angiosarcoma.

DFSP: dermatofibrosarcoma protuberans.

EpithelioidS: epithelioid sarcoma.

GIST: gastrointestinal stromal tumor.

KS: kaposi sarcoma.

LipoS: liposarcoma.

LMS: leiomyosarcoma.

MPNST: malignant peripheral nerve sheath tumor.

OsteoS: osteosarcoma.

SFT: solitary fibrous tumor.

SS: synovial sarcoma.

Unclassified S: unclassified sarcoma.

Among the 39 patients with GISTs, 31 were treated with sorafenib and the RR was 10% (3 PRs), three were treated with nilotinib, and three with the combination everolimus-cyclophosphamide. The other prescribed TTs for this histotype were masitinib (*n* = 1) and sunitinib-cyclophosphamide combination (*n* = 1). Among the 36 patients with non-uterine leiomyosarcoma (LMS), sorafenib was used in 22 cases with a RR of 14% (3 PRs), sunitinib in nine cases with a RR of 22% (2 PRs). The other patients were treated with everolimus (*n* = 3), imatinib (*n* = 1), and enzastaurin (*n* = 1). On the 18 angiosarcoma patients, 14 were treated with sorafenib with a RR of 21% (3 PRs), two were treated with sunitinib and the RR was 50% (1 PR). The remaining two patients were treated with bevacizumab-paclitaxel and sirolimus-cyclophosphamide combinations. Among the 15 patients with an unclassified sarcoma, eight were treated with sunitinib with a RR of 13% (1 PR), five were treated with sorafenib, one was treated with imatinib and one with panobinostat. Among 15 patients with chordoma, eight received imatinib, three received sunitinb, two received sorafenib, and one received sirolimus combined with cyclophosphamide. One patient with chordoma treated with erlotinib had a PR. Of 15 patients with osteosarcoma, ten were treated with sirolimus (including eight cases treated with sirolimus combined with cyclophosphamide), three were treated with sorafenib, and two with sunitinib. Among the 15 patients with synovial sarcoma (SS), eight were treated with sunitinib with a RR of 50% (4 PRs), three with sorafenib, two with pazopanib with a RR of 50% (1 PR), one with everolimus and one with cetuximab. For 125 patients with other histological subtypes comprising less than 15 patients each, TTs are described in Table 3.

**Table 3:** **Description of responses by histotypes and targeted therapy**

		TT1	n	%	CR	PR	RR (%)	TT2	n	%	CR	PR	RR (%)	Other PRs (n)
Total	278*				3	22					0	5		5
GIST	39	sorafenib	31	79	0	**3**	10	nilotinib	3	8	0	0	0	─
Leiomyosarcoma	36	sorafenib	22	61	0	**3**	14	sunitinib	9	25	0	**2**	22	─
Angiosarcoma	18	sorafenib	14	78	0	**3**	21	sunitinib	2	11	0	**1**	50	─
Unclassified sarcoma	15	sunitinib	8	53	0	**1**	13	sorafenib	5	33	0	0	0	─
Chordoma	15	imatinib	8	53	0	0	0	sunitinib	3	20	0	0	0	erlotinib (1)
Osteosarcoma	15	sirolimus	10	67	0	0	0	sorafenib	3	20	0	0	0	─
Synovialosarcoma	15	sunitinib	8	53	0	**4**	50	sorafenib	3	20	0	0	0	pazopanib (1)
Ewing / PNET	14	sunitinib	8	57	**1**	0	13	sorafenib	2	14	0	0	0	sirolimus + cyclop (1)
Chondrosarcoma	12	sirolimus	5	42	0	0	0	sorafenib	3	25	0	0	0	─
Uterine leiomyosarcoma	12	sorafenib	6	50	0	**1**	17	sunitinib	4	33	0	0	0	─
Liposarcoma	12	sorafenib	7	58	0	0	0	sunitinib	3	25	0	0	0	─
Solitary fibrous tumor	10	sorafenib	3	30	0	0	0	sunitinib	2	20	0	0	0	beva + TMZ (1)
Epithelioid sarcoma	9	sorafenib	2	22	0	**1**	50	sunitinib	2	22	0	0	0	beva + pacli (1)
MPNST	8	sorafenib	4	50	0	0	0	sunitinib	2	25	0	**1**	50	**─**
ASPS	8	sorafenib	5	63	0	**2**	40	sunitinib	3	38	0	0	0	─
DSRCT	6	sorafenib	3	50	0	0	0	sunitinib	3	50	0	0	0	─
AF	6	imatinib	4	67	0	0	0	sorafenib	1	17	0	**1**	100	─
DFSP	5	imatinib	4	80	**1**	**2**	75	sunitinib	1	20	0	0	0	─
PEComa	4	temsirolimus	2	50	**1**	**1**	100	sirolimus	1	25	0	0	0	─
Rhabdomyosarcoma	3	sunitinib	2	67	0	0	0	sorafenib	1	33	0	0	0	─
KS	1	everolimus	1	100	0	**1**	100	NA	─	─	─	─	─	─
Low grade ESS	1	sorafenib	1	100	0	0	0	NA	─	─	─	─	─	─
Phyllode tumor	1	sunitinib	1	100	0	0	0	NA	─	─	─	─	─	─
Other (including benign tumors)	13	ND	─	─	─	─		ND	─	─	─	─	─	─

Complete and partial responses are indicated in bold.

*represents the total number of treatment lines (some patients had several lines of targeted therapy).

TT1 is the most frequent targeted therapy, TT2 is the second most frequent targeted therapy.

AF: aggressive fibromatosis.

ASPS: alveolar soft parts sarcoma.

beva: bevacizumab.

CR: complete response.

cyclop: cyclophosphamide.

DFSP: dermatofibrosarcoma protuberans.

DSRCT: desmoplastic small round cell tumor.

ESS: endometrial stromal sarcoma.

GIST: gastro-intestinal stromal tumor.

KS: kaposi sarcoma.

MPNST: malignant peripheral nerve sheath tumor.

NA: not applicable.

ND: not described.

pacli: paclitaxel.

PR: partial response.

RR: response rate.

TMZ: temozolomide.

TT: targeted therapy.

More generally, the ORR of TTs for the whole group was 15% (95% CI [10,6-20,2]). Three patients had a CR: one patient with PEComa treated with temsirolimus who achieved a CR after seven months and stopped TT two months later because of continuing CR. The second patient was treated with sunitinib for a Ewing sarcoma. He stopped sunitinib after two months treatment. One patient with DFSP also achieved a CR with imatinib after five months of treatment. Thirty-two PRs were observed in 16 different subtypes with various TTs (17% of patients treated with sunitinib, 14% with sorafenib, 9% with imatinib, and 6% with sirolimus). CRs, PRs and corresponding TTs are described in Table 3. RRs superior to 20% were observed for DFSP with imatinib (75%, n = 4), for SS with sunitinib (50%, n = 8), for alveolar soft parts sarcoma with sorafenib (40%, n = 5), for non-uterine LMS with sunitinib (22%, n = 9), and also for angiosarcoma with sorafenib (21%, n = 14). Some other combinations showed signs of activity, but with two patients or less per group (described in Table 3). The DCR was 59% at two months, 39% at four months, and 25% at six months.

### Follow-up and survival

**Figure 1:**
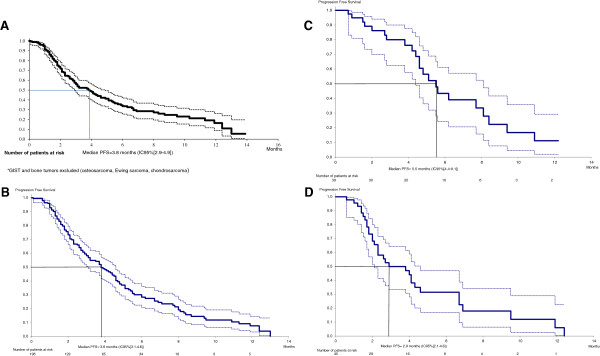
**Progression-free survival data. A**. Progression-free survival for soft-tissue sarcoma (except GIST). **B**: Progression-free survival of the patients in third line and after (number of previous chemotherapy >2). **C**: Progression-free survival of the GIST group. **D**: Progression-free survival of the leiomyosarcoma group.

Median follow-up since diagnosis was five years (range: 0,1 to 32,9, *n* = 234, 14 patients without follow-up data and one patient with missing date of diagnosis). The median PFS of the entire series was 4,1 months (95% CI [3,2-4,8]). For STS (Figure 1A), median PFS was 3,8 months (95% CI [2,4,9]). Figure 1B shows the PFS of patients treated with TTs in third line and beyond. GIST and LMS, the most two frequent subtypes, had a median PFS of 5,5 months (95% CI [4,4-8,1]) and 2,9 months (95% CI [2,1-4,6]) respectively (Figure 1C and D). Forty-eight patients died (20%), of underlying cancer in 98% of cases (47 patients). One patient died from a massive pulmonary embolism during treatment with sunitinib, in the context of an intra-cardiac metastasis of a high-grade undifferentiated thoracic sarcoma (this event was not attributed to sunitinib by the investigator).

### Toxicity of targeted therapies (Table 4)

**Table 4:** **Toxicities**

	Total*	GRADE**			
	N (%)	1 N (%)	2 N (%)	3 N (%)	4 N (%)
SORAFENIB (n = 116)					
At least 1 toxicity reported	94 (81)	─	─	─	─
Intestinal toxicity	63 (54)	26 (22)	27 (23)	13 (11)	0 (0)
Skin toxicity, infections	56 (49)	26 (22)	20 (18)	12 (10)	0 (0)
Hematologic toxicity	18 (16)	5 (4)	10 (9)	5 (4)	0 (0)
Neurologic toxicity	12 (10)	9 (3)	2 (2)	1 (1)	0 (0)
Cardiologic toxicity	10 (9)	4 (3)	4 (3)	2 (2)	0 (0)
Pulmonary toxicity	4 (3)	2 (2)	1 (1)	1 (1)	0 (0)
Renal toxicity	1 (1)	0 (0)	1 (1)	0 (0)	0 (0)
Other toxicities	67 (58)	19 (16)	34 (29)	19 (16)	1 (1)
SUNITINIB (n = 66)					
At least 1 toxicity reported	49 (74)	─	─	─	─
Intestinal toxicity	26 (39)	16 (24)	7 (11)	5 (8)	0 (0)
Hematologic toxicity	21 (32)	5 (8)	11 (17)	3 (5)	4 (6)
Skin toxicity, infections	12 (18)	6 (9)	6 (9)	1 (2)	0 (0)
Neurologic toxicity	6 (9)	2 (3)	1 (2)	2 (3)	1 (2)
Pulmonary toxicity	5 (8)	3 (5)	1 (2)	1 (2)	0 (0)
Cardiologic toxicity	3 (5)	3 (5)	0 (0)	0 (0)	0 (0)
Renal toxicity	1 (2)	0 (0)	0 (0)	0 (0)	1 (2)
Other toxicities	38 (58)	15 (23)	14 (21)	13 (20)	2 (3)
IMATINIB (n = 23)					
At least 1 toxicity reported	20 (87)	─	─	─	─
Intestinal toxicity	12 (52)	6 (26)	4 (17)	1 (4)	1 (4)
Hematologic toxicity	6 (26)	2 (9)	2 (9)	2 (9)	0 (0)
Skin toxicity, infections	2 (9)	2 (9)	0 (0)	0 (0)	0 (0)
Neurologic toxicity	1 (4)	0 (0)	1 (4)	1 (4)	0 (0)
Pulmonary toxicity	0 (0)	0 (0)	0 (0)	0 (0)	0 (0)
Cardiologic toxicity	0 (0)	0 (0)	0 (0)	0 (0)	0 (0)
Renal toxicity	0 (0)	0 (0)	0 (0)	0 (0)	0 (0)
Other toxicities	17 (74)	4 (17)	12 (52)	3 (13)	0 (0)
SIROLIMUS + CYCLOPHOSPHAMIDE (n = 18)					
At least 1 toxicity reported	9 (50)	─	─	─	─
Intestinal toxicity	5 (28)	4 (22)	1 (6)	0 (0)	0 (0)
Hematologic toxicity	3 (17)	1 (6)	1 (6)	0 (0)	1 (6)
Pulmonary toxicity	2 (11)	1 (6)	0 (0)	0 (0)	1 (6)
Skin toxicity, infections	1 (6)	1 (6)	0 (0)	0 (0)	0 (0)
Neurologic toxicity	1 (6)	0 (0)	1 (6)	0 (0)	0 (0)
Renal toxicity	1 (6)	0 (0)	0 (0)	1 (6)	0 (0)
Cardiologic toxicity	0 (0)	0 (0)	0 (0)	0 (0)	0 (0)
Other toxicities	7 (39)	3 (17)	4 (22)	0 (0)	0 (0)

*number of lines of treatment.

**below are described the numbers and percentages of patients with at least one toxicity of each grade.

(a patient could have experienced several grades for the same type of toxicity).

Among the 278 lines of treatment, 208 (75%) patients developed at least one AE during the follow-up. Gastro-intestinal toxicity was observed in 125 (45%) patients: 25% patients had diarrhea, 9% stomatitis, 5% nausea, 5% vomiting, and 3% anorexia. Skin toxicities and hematologic toxicities were present in 29 and 22% of patients respectively. Other side effects (pulmonary, cardiac, and neurologic) were rare and observed in less than 10% of cases. These AEs were mostly grade 1–2 (42% of patients). Grade 3 toxicities were observed for 74 patients (27%) and were gastro-intestinal (8%) hematologic (5%) and skin/infectious toxicities (5%). Only 14 patients (5%) experienced grade 4 toxicity, in the majority of cases these were hematologic toxicities (6 patients, 2%). As expected, sorafenib, sunitinib and imatinib had different toxicity profiles, but overall, the rate of grade 3–4 AE was similar between these three agents (36% with sorafenib, 40% with sunitinib and 35% with imatinib). No toxic death occurred during follow-up.

## Discussion

### Why off label use and how it is selected

Designing and conducting prospective clinical trials in very rare tumors is challenging. After standard treatments, cytotoxic chemotherapies are often used in routine practice on the basis of phase II trials without evidence from randomized studies [10, 11]. We anticipated that results of different phase II trials of TT in different sarcomas would lead to similar off-label use, and decided to analyze their impact on survival, response rate and toxicities within a prospective registry.

In term of routine practice, OUTC’S program confirmed that most patients received TTs that have already shown signs of activity in phase II clinical trials. More than 74 therapeutic combinations were proposed. 38% of the decisions were based on published data, 30% on personal communications, and 29% on biological hypothesis (Additional file 2: Table S2). Only 4% of TT treatments had no scientific rationale. Similarly, treatment decision was made mostly following discussion at MSB, which works on a careful evaluation of benefit/risk balance for these off-label TTs. Functioning and organization of MSB in France seem comparable to other countries [32]; these data could therefore be extrapolated elsewhere.

### Efficacy and safety of treatment

DCR was 59% at 2 months, median PFS was 4,1 months, and 3,8 months when considering the STS group. This confirms that off-label TTs can be considered as active treatments, according to the analysis reported by the EORTC Soft Tissue and Bone Sarcoma Group [33]. Median OS is not interpretable due to the group heterogeneity, and for the same reason, median PFS should be interpreted with caution. Three CRs and 32 PRs were observed. The efficacy of off-label TTs in this study is comparable to that reported in previous publications: in a phase II study, Maki and colleagues observed a median PFS of 3,2 months with sorafenib as a single agent in patients with recurrent or metastatic STS [22]. Another phase II study with 48 patients with non-GIST sarcomas reported one PR and ten SD at 16 weeks with sunitinib single agent [23]. In PALETTE phase III study, patients receiving pazopanib had a median PFS of 4,6 months. All these patients had a metastatic soft-tissue sarcoma, progressing despite previous standard chemotherapy and had received up to 3 lines of prior treatment [24]. Median PFS of the LMS group was 2,9 months here. As a comparison, in the phase II study by Mahmood et al., LMS patients treated with sunitinib had a median PFS of 4,2 months [34]. The RR of GIST-patients treated with sorafenib was 10% in our population, compared to 13% in the phase II trial by Kindler and colleagues [35]. In the sunitinib phase II trial [23], among four patients with SS, only one had a SD as best response. Here, among eight patients treated with sunitinib for a SS, four had a PR, corresponding to a RR of 50%. Hence, the analysis of this registry confirms that TTs with activity in phase II trials can also demonstrate activity of a similar magnitude in an off-label setting, in a less selected population. Moreover, the population included in clinical trials often received fewer prior systemic therapies. For example, patients included in the sunitinib trial had only received two lines of treatment before inclusion [23], and only 63% of patients had one or more prior line chemotherapy in the sorafenib trial [22]. The reported AEs (mostly grade I or II) were similar to those reported in clinical trials [22, 23], even in this non-selected population.

### A useful method to collect information for off label treatment

This study shows an original methodological approach to collect information for off-label use of TT in rare tumors, for patients who cannot be included in clinical trials, either because of inappropriate inclusion criteria or because clinical trials do not exist. Some histological subtypes are too rare to get pharmaceutical companies interested in developing randomized trials. The major limitation of this study is that it is only descriptive, not randomized and therefore lacks a control arm. However, it confirms safety of TTs in routine practice, and has practical interest, in particular for therapeutic niches. The latest developments in Ewing’s sarcoma tend to show that Insulin-like Growth Factor 1 Receptor (IGF1R) targeting agents are promising [36, 37], but none was efficient enough to lead to a phase III trial. Our case of CR with sunitinib opens the way to the exploration of VEGFR and PDGFR pathways in Ewing’s sarcoma. The inactivation of TSC1/TSC2 in PEComa leads to increased mTOR complex 1 (TORC1) activation [36, 38]. In other reports [39, 40], three patients treated with sirolimus achieved PR, one patient had a PR with temsirolimus and another one had a CR with the same rapalog. Even if resistance to mTOR targeting agent has been reported in this very rare pathology [41], this new case of CR in the OUTC’S program with an mTOR inhibitor is encouraging to engage in a prospective clinical trial.

Collection of additional molecular data was not planned as part of this study. Patients were included between 2008 and 2011, and very few molecular biology platforms were able to perform tumor-sequencing analysis routinely at this time. The question of treating patients according to actionable genomic alterations in advanced malignancies is currently being addressed in specific clinical trials, such as the Profiler [42] and SHIVA [43] trials in France.

## Conclusions

In conclusion, this registry collected data on a large number of patients treated with off-label TTs in various sarcoma subtypes. No major toxic or unusual side effects were observed and efficacy was similar to that observed in published trials. Discussion of cases by a MSB to determine the legitimacy of such treatments ensures some consensus among experts in the field, and gives the opportunity for heavily pre-treated patients access these new agents. This methodological approach could be easily extrapolated to other rare cancers, in the absence of clinical trials.

## Electronic supplementary material

Additional file 1: **Participating centers.** (DOCX 60 KB)

Additional file 2: Table S1: First line therapy. **Table S2.** rationale of the targeted therapy prescription. (DOCX 40 KB)
