# Supplementary material for: The off-label use of targeted therapies in sarcomas: the OUTC’S program
Source: BMC Cancer. 2014 Nov 24;14:870. doi: 10.1186/1471-2407-14-870 (PMC4289372; doi:10.1186/1471-2407-14-870)
Supplement: Supplementary file 1 — Additional file 1: Participating centers. (DOCX 60 KB) [file 12885_2014_5121_MOESM1_ESM.docx]

MS: 1375378806128305

**The off-label use of targeted-therapies in sarcomas: the OUTC'S program.**

Lauriane Eberst, Claire Cropet, Axel Le Cesne, Patricia Pautier, Nicolas Penel, Antoine Adenis, Christine Chevreau, Jacques-Olivier Bay, Olivier Collard, Didier Cupissol, Florence Duffaud, Jean-Claude Gentet, Sophie Piperno-Neumann, Perrine Marec-Bérard, Emmanuelle Bompas, Antoine Thyss, Loic Chaigneau, Philippe Cassier, François Bertucci, Jean-Yves Blay, and Isabelle Ray-Coquard.

**Participating centers :**

Centre Léon Bérard and Institut d’Hématologie et d’Oncologie Pédiatrique, Lyon ; Institut Gustave Roussy, Paris ; Institut Bergonié, Bordeaux ; Institut Paoli Calmettes, Marseille ; Centre Oscar Lambret, Lille ; Centre René Gauducheau, Nantes ; Centre Eugène Marquis, Rennes ; Centre Antoine Lacassagne, Nice ; Centre François Baclesse, Caen; Centre Val d’Aurelle, Montpellier; Institut Curie, Paris ; Institut de Cancérologie Lucien Neuwirth, Saint Etienne; Centre René Huguenin, Saint Cloud ; Centre Alexis Vautrin, Nancy ; Institut Claudius Regaud, Toulouse ; Centre Jean Perrin, Clermont-Ferrand ; Centre Henri-Becquerel, Rouen ; Cochin Hospital, Paris ; Hotel Dieu and Mother and Child Hospital, Nantes ; Edouard Herriot Hospital, Lyon ; Henri Mondor Hospital, Créteil ; La Timone Hospital, Marseille; Saint Louis Hospital, Paris ; Raymond Poincaré Hospital, Garches ; Jean Minjoz Hospital, Besançon.
