# Supplementary material for: The off-label use of targeted therapies in sarcomas: the OUTC’S program
Source: BMC Cancer. 2014 Nov 24;14:870. doi: 10.1186/1471-2407-14-870 (PMC4289372; doi:10.1186/1471-2407-14-870)
Supplement: Supplementary file 2 — Additional file 2: Table S1: First line therapy. Table S2. rationale of the targeted therapy prescription. (DOCX 40 KB) [file 12885_2014_5121_MOESM2_ESM.docx]

**THE OFF-LABEL USE OF TARGETED THERAPIES IN SARCOMAS: THE OUTC’S PROGRAM.**

**SUPPLEMENTARY DATA**

**Table 1: First line therapy**

|  |  |  |  |  |
| --- | --- | --- | --- | --- |
|  | Surgery of primary site | **N=278** |  |  |
|  | Unknown (%) | 1 | (0.4) |  |
|  | No (%) | 65 | (23.5) |  |
|  | Yes (%) | 212 | (76.5) |  |
|  | Quality of resection | **N=212** |  |  |
|  | Unknown (%) | 45 | (21.2) |  |
|  | R0 (%) | 81 | (48.5) |  |
|  | R1 (%) | 52 | (31.1) |  |
|  | R2 (%) | 34 | (20.4) |  |
|  | First line treatment* | **N=278** |  |  |
|  | First line radiotherapy |  |  |  |
|  | No (%) | 200 | (71.9) |  |
|  | Yes (%) | 78 | (28.1) |  |
|  | First line chemotherapy |  |  |  |
|  | No (%) | 70 | (25.2) |  |
|  | Yes (%) | 208 | (74.8) |  |
|  | First line TT |  |  |  |
|  | No (%) | 262 | (94.2) |  |
|  | TT alone (%) | 15 | (5.4) |  |
|  | Chemotherapy + TT [1] (%) | 1 | (0.4) |  |
|  | Response at the end of first line therapy (N=278) | |  |  |
|  | No evidence of progression (%) | 149 | (53.6) |  |
|  | Progressive patients (%) | 76 | (29.6) |  |
|  | Non evaluable (%) | 32 | (12.5) |  |
|  | Unknown (%) | 21 | (7.6) |  |
|  |  |  |  |  |
|  |  |  |  |  |
|  | *patients could have either/and radiation, chemotherapy, TT | |  |  |
|  |  |  |  |  |
|  | TT: targeted therapy |  |  |  |
|  | [1]: sirolimus+cyclophosphamide |  |  |  |
|  |  |  |  |  |

**Table 2 : rationale of the targeted therapy prescription**

|  |  |  |  |  |  |  |  |  |  |
| --- | --- | --- | --- | --- | --- | --- | --- | --- | --- |
|  | **Histology** | **TT** | **n** | **Publication (type)*** | **If no publication, oral communication (type)**** | **If no oral communication, biological hypothesis***** | **No scientific rationale§** |  |  |
|  | Aggressive fibromatosis | imatinib | 4 | **Yes (ph II)** | - | - | - |  |  |
|  | n=6 | sorafenib | 1 | No | No | **Yes** | - |  |  |
|  |  | sunitinib | 1 | **Yes (CR)** | - | - | - |  |  |
|  | Angiosarcoma | bevacizumab | 1 | **Yes (CR)** | - | - | - |  |  |
|  | n=18 | sirolimus | 1 | **Yes (CR)** | - | - | - |  |  |
|  |  | sorafenib | 14 | **Yes (CR)** | - | - | - |  |  |
|  |  | sunitinib | 2 | **Yes (CR)** | - | - | - |  |  |
|  | ASPS | sorafenib | 5 | No | No | **Yes** | - |  |  |
|  | n=8 | sunitinib | 3 | **Yes(ph II)** | - | - | - |  |  |
|  | Chondrosarcoma | sirolimus | 6 | No | **Yes (ASCO 2011)** | - | - |  |  |
|  | n=12 | imatinib | 1 | No | **Yes (ASCO 2010)** | - | - |  |  |
|  | (1 pt with zoledronate) | sorafenib | 3 | No | No | **Yes** | - |  |  |
|  |  | sunitinib | 1 | **Yes (CR)** | - | - | - |  |  |
|  | Chordoma | erlotinib | 1 | **Yes (CR)** | - | - | - |  |  |
|  | n=15 | imatinib | 8 | **Yes (CR)** | - | - | - |  |  |
|  |  | sirolimus | 1 | **Yes (CR)** | - | - | - |  |  |
|  |  | sorafenib | 2 | No | No | No | **Yes** |  |  |
|  |  | sunitinib | 3 | No | No | No | **Yes** |  |  |
|  | DFSP | imatinib | 4 | **Yes (approvedI)** | - | - | - |  |  |
|  | n=5 | sunitinib | 1 | **No** | No | **Yes** | - |  |  |
|  | DSRCT | sorafenib | 3 | No | No | **Yes** | - |  |  |
|  | n=6 | sunitinib | 3 | No | No | **Yes** | - |  |  |
|  | Epithelioid sarcoma | bevacizumab | 1 | No | No | **Yes** | - |  |  |
|  | n=9 | imatinib | 2 | No | No | No | **Yes** |  |  |
|  |  | sorafenib | 4 | **Yes (ph II)** | - | - | - |  |  |
|  |  | sunitinib | 2 | No | No | **Yes** | - |  |  |
|  | Ewing'-PNET | enzastaurin | 1 | No | No | No | **Yes** |  |  |
|  | n=14 | sirolimus | 3 | No | No | **Yes** | - |  |  |
|  |  | sorafenib | 2 | No | No | **Yes** | - |  |  |
|  |  | sunitinib | 8 | No | No | **Yes** | - |  |  |
|  | GIST | sirolimus | 3 | No | **Yes (ASCO 2009)** | - | - |  |  |
|  | n=39 | masitinib | 1 | **Yes (ph II)** | - | - | - |  |  |
|  |  | sunitinib | 1 | **Yes (approved)** | - | - | - |  |  |
|  |  | sorafenib | 31 | No (not in 2012) | **Yes (ASCO 2009)** | - | - |  |  |
|  |  | nilotinib | 3 | No (not in 2012) | **Yes (ASCO 2008)** | - | - |  |  |
|  | Kaposi sarcoma | sirolimus | 1 | No | No | **Yes** | - |  |  |
|  | Liposarcoma | sirolimus | 1 | No | No | **Yes** | - |  |  |
|  | n=12 | sorafenib | 8 | No | No | **Yes** | - |  |  |
|  |  | sunitinib | 3 | **Yes (ph II)** | - | - | - |  |  |
|  | LMS | sirolimus | 3 | No | No | **Yes** | - |  |  |
|  | n=36 | enzastaurin | 1 | No | No | **Yes** | - |  |  |
|  |  | imatinib | 1 | No | No | **Yes** | - |  |  |
|  |  | sorafenib | 22 | No | **Yes (ASCO 2011)** | - | - |  |  |
|  |  | sunitinib | 9 | **Yes (ph II)** | - | - | - |  |  |
|  | Low grade ESS | sorafenib | 1 | No | No | No | **Yes** |  |  |
|  | MPNST | sirolimus | 2 | No | No | **Yes** | - |  |  |
|  | n=8 | sorafenib | 4 | **Yes (ph II)** | **Yes (ASCO 2011)** | - | - |  |  |
|  |  | sunitinib | 2 | **Yes (ph II)** | No | **Yes** | - |  |  |
|  | Osteosarcoma | sirolimus | 10 | **Yes (ph II)** | - | **Yes** | - |  |  |
|  | n=15 | sorafenib | 3 | No | No | **Yes** | - |  |  |
|  |  | sunitinib | 2 | No | No | **Yes** | - |  |  |
|  | PEComa | sirolimus | 3 | **Yes (CR)** | - | - | - |  |  |
|  | n=4 | sunitinib | 1 | No | No | **Yes** | - |  |  |
|  | Phyllode tumor | sunitinib | 1 | No | No | **Yes** | - |  |  |
|  | Rhabdomyosarcoma | sorafenib | 1 | No | No | **Yes** | - |  |  |
|  | n=3 | sunitinib | 2 | No | No | **Yes** | - |  |  |
|  | Solitary fibrous tumor | bevacizumab | 2 | No | No | **Yes** | - |  |  |
|  |  | sirolimus | 2 | **Yes (ph II)** | - | - | - |  |  |
|  | n=10 | imatinib | 1 | **Yes (ph II)** | - | - | - |  |  |
|  |  | sorafenib | 3 | **Yes (ph II)** | - | - | - |  |  |
|  |  | sunitinib | 2 | No | **Yes (2009)** | - | - |  |  |
|  | Synovial sarcoma | cetuximab | 1 | No | No | **Yes** | - |  |  |
|  | n=15 | sirolimus | 1 | No | No | **Yes** | - |  |  |
|  |  | pazopanib | 2 | No | No | **Yes** | - |  |  |
|  |  | sorafenib | 3 | No | **Yes (ASCO 2011)** | **-** | - |  |  |
|  |  | sunitinib | 8 | **Yes (ph II)** | - | - | - |  |  |
|  | Unclassified | enzastaurin | 1 | No | No | No | **Yes** |  |  |
|  | n=15 | imatinib | 1 | No | No | No | **Yes** |  |  |
|  |  | sorafenib | 5 | **Yes (ph II)** | No | No | - |  |  |
|  |  | sunitinib | 8 | **Yes (ph II)** | No | No | - |  |  |
|  | Uterine LMS | sorafenib | 8 | No | **Yes (ASCO 2011)** | - | - |  |  |
|  | n=12 | sunitinib | 4 | **Yes (ph II)** | - | - | - |  |  |
|  | Other histologies are not described here (n=12). Some of the TTs were prescribed in association with chemotherapy : in this case, only the TT was selected to analyze the rationale. | | | | | | | |  |
|  | * before 2012, in Pubmed | | | | | | | |  |
|  | ** in an international cancer congress | | | | | | | |  |
|  | *** research article in Pubmed | | | | | | | |  |
|  | § if previous criteria were not met | | | | | | | | |
|  |  | | | | | | | | |
|  | ASPS: alveolar soft parts sarcoma | | | | | | | | |
|  | ASCO: american society of clinical oncology | | | | | | | | |
|  | CR: case-report | | | | | | | | |
|  | DFSP: Dermtofibrosarcoma Protuberans | | | | | | | | |
|  | DSRCT: desmoplastic small round cell tumor | | | | | | | | |
|  | ESS: endometrial stromal tumor | | | | | | | | |
|  | LMS: leiomyosarcoma | | | | | | | | |
|  | MPNST: malignant peripheral nerve sheath tumor | | | | | | | | |
|  | phII: phase II | | | | | | | | |
|  | pt: patient | | | | | | | | |
